# Comparison of Pyrazinamide with Isoniazid for Their Effects on the Heme Biosynthetic Pathway in Mouse Liver

**DOI:** 10.3390/metabo15060355

**Published:** 2025-05-28

**Authors:** Fu-Ying Qin, Ruizhi Gu, Jiaojiao Zhang, Jaden Leigh Weiss, Jie Lu, Qing Ma, Xiaochao Ma

**Affiliations:** 1 Center for Pharmacogenetics, Department of Pharmaceutical Sciences, School of Pharmacy, University of Pittsburgh, 335 Sutherland Drive, Pittsburgh, PA 15261, USA; fuq1@pitt.edu (F.-Y.Q.); rug25@pitt.edu (R.G.); jiz474@pitt.edu (J.Z.); jlw222@pitt.edu (J.L.W.); jil151@pitt.edu (J.L.); 2 Department of Pharmacy Practice, School of Pharmacy and Pharmaceutical Sciences, University at Buffalo, Buffalo, NY 14214, USA; qingma@buffalo.edu

**Keywords:** pyrazinamide, isoniazid, aminolevulinic acid synthase, ferrochelatase, porphyria

## Abstract

**Background/Objectives:** Isoniazid (INH) and pyrazinamide (PZA) are first-line drugs used to treat tuberculosis (TB), but their use is generally contraindicated in patients with porphyria, a group of metabolic disorders caused by defects in the heme biosynthetic pathway. To investigate the basis for these contraindications, we compared the effects of INH and PZA on the heme biosynthetic pathway in mouse liver. **Method:** We investigated the hepatic expression and activity of the key enzymes involved in the heme biosynthetic pathway, including aminolevulinic acid synthase 1 (Alas1) and ferrochelatase (Fech). Additionally, we employed a metabolomic approach to analyze liver and fecal samples from the mice treated with INH or PZA. **Result:** We found that INH, but not PZA, significantly upregulated the expression and activity of Alas1, the rate-limiting enzyme in heme biosynthesis, while concurrently downregulating Fech, which converts protoporphyrin IX (PPIX) to heme. These changes resulted in the accumulation of the toxic intermediate aminolevulinic acid (ALA) and PPIX in the liver of INH-treated mice. In contrast, PZA had no measurable effect on the expression or function of Alas1 or Fech. **Conclusions:** These findings provide mechanistic insight into INH-induced porphyria exacerbation and suggest that PZA may not carry the same risk, challenging its current contraindication.

## 1. Introduction

Tuberculosis (TB) is an infectious disease caused by *Mycobacterium tuberculosis* (Mtb), affecting approximately 10.8 million people worldwide and leading to 1.25 million deaths in 2023 [1]. Pyrazinamide (PZA) and isoniazid (INH) are first-line drugs for the treatment of TB. INH is a prodrug that is activated by the mycobacterial catalase-peroxidase enzyme KatG to form an isonicotinyl-nicotinamide adenine dinucleotide adduct, which inhibits Mtb’s cell wall biosynthesis [2,3,4]. PZA shares structural similarity with INH, and its introduction into the TB drug regimen has shortened the treatment from 12 to 6 months. Like INH, PZA is also a prodrug, which is converted into its bioactive metabolite, pyrazinoic acid (PA), by pyrazinamidase [5,6]. Aspartate decarboxylase, an enzyme essential for coenzyme A biosynthesis, has been identified as a key target of PA [7,8]. While PZA and INH play important roles in the treatment of TB, both are contraindicated in patients with porphyria [9,10,11,12,13], and the underlying mechanisms behind these contraindications remain unclear.

Porphyria comprises a group of metabolic disorders resulting from defects in the heme biosynthetic pathway [14,15]. This pathway involves eight enzymatic steps: aminolevulinic acid synthase (ALAS), aminolevulinic acid dehydratase, porphobilinogen deaminase, uroporphyrinogen III synthase, uroporphyrinogen decarboxylase, coproporphyrinogen oxidase, protoporphyrinogen oxidase, and ferrochelatase (FECH). Among these enzymes, ALAS catalyzes the first and rate-limiting step, producing aminolevulinic acid (ALA) [16], while FECH catalyzes the final step, converting protoporphyrin IX (PPIX) into heme [17]. Given the critical roles of ALAS and FECH in the heme biosynthetic pathway, these enzymes serve as key targets for investigating drug-induced disturbances in heme metabolism.

Previous studies using animal models have demonstrated that INH interacts with both Alas and Fech, key enzymes in the heme biosynthetic pathway [18,19], supporting the recommendation that INH should be avoided in patients with porphyria. In contrast, data on PZA and its impact on this pathway remain limited. In the 1960s, one study reported that PZA induced Alas activity in primary cultures of chick embryo liver cells grown on cover slips [20]. A follow-up study in the 1970s observed increased hepatic Alas activity in rats following a single dose of PZA, an effect not seen in the INH-treated group [21]. However, these early studies are limited in several key aspects. They primarily assessed Alas activity in isolated cell models [20], which may not fully capture the complexity of the heme biosynthetic pathway in vivo. Moreover, they evaluated only the effects of a single dose of PZA, whereas clinical regimens for TB involve chronic exposure, which may lead to different outcomes [21]. Because both INH and PZA are contraindicated in porphyria but remain essential components of TB treatment regimens [9,10,11,12,13], it is critical to elucidate the underlying mechanisms of these contraindications and to develop novel strategies for the prevention of such adverse effects.

To address the aforementioned knowledge gaps, we systematically investigated the acute and subchronic effects of INH and PZA on the expression and function of Alas and Fech in the mouse liver. Our findings provide novel insights into the molecular impact of these drugs and offer important guidance for the safer use of INH and PZA in TB patients with porphyria.

## 2. Materials and Methods

### 2.1. Chemicals and Reagents

PZA, INH, ALA, ^15^N,^13^C_5_-aminolevulinic acid (^15^N,^13^C_5_-ALA), and PPIX were purchased from Sigma-Aldrich (St. Louis, MO, USA). The AccQ Tag derivatization kit was purchased from Waters (Milford, MA, USA). The solvents for metabolite analysis, including acetonitrile and water, were purchased from Fisher Scientific (Fair Lawn, NJ, USA).

### 2.2. Animals and Treatment

Wild-type (WT, C57BL/6) mice (6 weeks-old, male) were obtained from the Jackson Laboratory (Bar Harbor, ME, USA) and maintained on a 12 h light/dark cycle with water and chow provided ad libitum. The mice were treated with PZA (2000 mg/L) or INH (400 mg/L) in drinking water for 14 days. Afterward, liver and blood samples were collected for further analysis. Another three groups of WT mice were treated orally with a single dose of vehicle, PZA (360 mg/kg), or INH (72 mg/kg) and housed separately in metabolic cages overnight to collect urine and feces. The animal studies were approved by the Institutional Animal Care and Use Committee of the University of Pittsburgh.

### 2.3. Quantitative Polymerase Chain Reaction (qPCR) Analysis

Total mRNA was extracted from the liver by using a TRIzol reagent (Invitrogen, Carlsbad, CA, USA). Complementary DNA (cDNA) was generated from 1 μg of total RNA using a SuperScript II Reverse Transcriptase kit and random oligonucleotides (Invitrogen). A qPCR analysis was conducted using 25 ng cDNA, 150 nM of each primer, and 5 μL of SYBR Green PCR Master Mix (Applied Biosystems, Foster City, CA, USA) in a total volume of 10 μL. The primers for the qPCR analysis of Alas1 were TCGCCGATGCCCATTCTTATC (forward) and GGCCCCAACTTCCATCATCT (reverse). The primers for the qPCR analysis of Fech were CAGACAGATGAGGCTATCAAAGG (forward) and CACAGCTTGTTGGACTGGATG (reverse). The qPCR plate was read on an ABI-Prism 7500 Sequence Detection System (Applied Biosystems, Foster City, CA, USA) and quantified using the comparative CT method.

### 2.4. Western Blotting Analysis

Mouse liver homogenate was prepared for Western blotting of Alas1 and Fech. Briefly, and protein concentrations of mouse liver homogenate were determined using the Pierce BCA Protein Assay Kit (ThermoFisher, Carlsbad, CA, USA). Twenty μg of protein from each sample was resolved on a 10% SDS-polyacrylamide gel. Afterward, the proteins were transferred to PVDF membranes. The membranes were incubated with primary antibodies against Alas1 (Abcam, ab84962), Fech (Santa Cruz, sc-377377), and Gapdh (EMD Millipore, MAB374). Immunoreactive proteins were identified by a chemiluminescence method.

### 2.5. ALA Analysis

ALA was analyzed in the liver and urine, a site for production and the route for excretion of ALA, respectively [16,22,23]. In brief, liver samples were homogenized in water (100 mg of tissues in 250 µL of water), and then, 200 µL of acetonitrile/methanol (1:1, *v*/*v*) was added to 100 µL of liver homogenate, followed by vortex and centrifugation at 15,000× *g* for 10 min. For the urine samples, 20 µL of urine was added to 180 µL of acetonitrile/methanol/water (4:4:1, *v*/*v*/*v*) and then vortexed and centrifuged at 15,000× *g* for 10 min. The derivatization of ALA was carried out following the instructions of the AccQ Tag kit. ¹⁵N, ^13^C₅-ALA was used as a positive control for ALA analysis. Derivatized ALA and ¹⁵N, ^13^C₅-ALA were analyzed by ultra-performance liquid chromatography–quadrupole time of flight mass spectrometry (UPLC-QTOFMS, Waters, Milford, MA, USA).

### 2.6. PPIX Analysis

PPIX was analyzed in the liver and feces because PPIX is mainly excreted through the hepatobiliary system as feces [22,24]. The method for the extraction of PPIX from the liver samples was the same as that in a previous report [25]. For the fecal samples, 100 mg of feces was homogenized in 1000 µL of water, and then, 300 µL of acetonitrile/methanol (1:1, *v*/*v*) was added to 100 µL of fecal homogenate, followed by vortexing and centrifugation at 15,000× *g* for 10 min. Each supernatant was transferred to an autosampler vial, and 2.0 µL was injected into the UPLC-QTOFMS system for metabolite analysis.

### 2.7. UPLC-QTOFMS Analysis

Metabolite separation was performed using an ACQUITY HSS T3 column (Waters, Milford, MA, USA). The mobile phase flow rate was 0.5 mL/min, with a gradient ranging from 2% to 95% acetonitrile/water containing 0.1% formic acid. The temperature of the column was maintained at 45 °C. QTOFMS was operated in positive mode with electrospray ionization. The source and desolvation temperatures were set to 150 °C and 500 °C, respectively. Nitrogen was used as both the cone and desolvation gas. The capillary and cone voltages were set at 0.8 kV and 40 V, respectively. MS data (50–1000 Da) were acquired in centroid mode using MassLynx 4.1 software (Waters, Milford, MA, USA).

### 2.8. Metabolomic Analysis

After the UPLC-QTOFMS analysis of liver and fecal samples, a multivariate data matrix containing sample identity, ion features (*m*/*z* and retention time), and ion abundance was generated and exported to SIMCA-P+ (Version 13, Umetrics, Kinnelon, NJ, USA) for further analysis. Briefly, principal component analysis (PCA) and orthogonal partial least-squares discriminant analysis (OPLS-DA) were performed to evaluate metabolic differences among sample groups.

### 2.9. Assessment of Liver Injury

The serum levels of alanine transaminase (ALT), aspartate transaminase (AST), and alkaline phosphatase (ALP) were measured using standard assay kits according to the manufacturer’s instructions (Point Scientific Inc., Canton, MI, USA). Additionally, liver tissues were fixed in 4% phosphate-buffered formaldehyde overnight, then dehydrated, and embedded in paraffin. Sections of 4 µm thickness were cut and stained with hematoxylin and eosin (H&E) for histological analysis.

### 2.10. Statistical Analysis

The data are expressed as means ± S.D. Statistical analyses were performed using one-way ANOVA. *p* < 0.05 was considered statistically significant.

## 3. Results

### 3.1. The Exposure of PZA and INH to Mice

**Figure 1:**
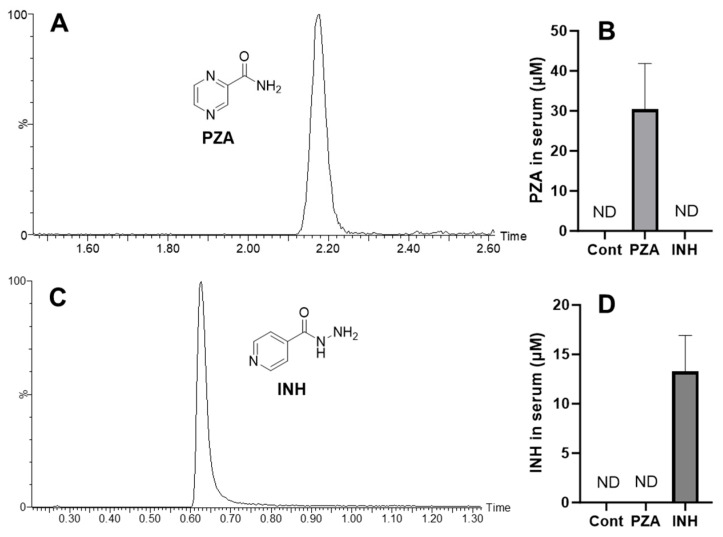
Detection of PZA and INH in serum. WT mice were treated with 2000 mg/L PZA or 400 mg/L INH in drinking water for 14 days. Serum samples were analyzed by UPLC-QTOFMS. (**A**) UPLC spectrum of PZA. (**B**) Quantification of PZA in serum. (**C**) UPLC spectrum of INH. (**D**) Quantification of INH in serum. Data are expressed as mean ± SD (*n* = 4). ND, not detected.

WT mice were treated with PZA or INH via drinking water for 14 days. Drug exposure levels were confirmed by measuring serum concentrations using a UPLC-QTOFMS analysis. PZA exhibited a retention time of 2.18 min, with a corresponding serum concentration of 30.4 µM (Figure 1A,B). PA, the active form of PZA, was also detected in the serum of mice treated with PZA (Supplementary Figure S1). In addition, INH showed a retention time of 0.63 min, and its serum concentration was 13.3 µM (Figure 1C,D).

### 3.2. Effects of PZA and INH on Alas1 Expression in the Liver

**Figure 2:**
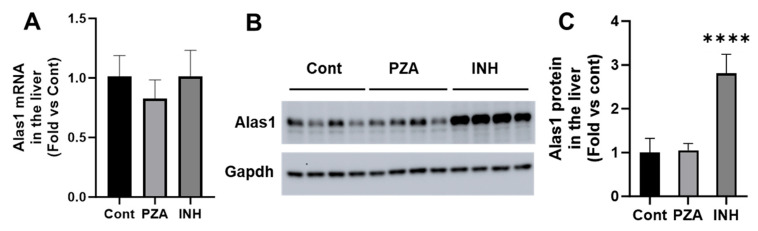
Effects of PZA and INH on Alas1 expression in the liver. WT mice were treated with PZA or INH for 14 days. (**A**) qPCR analysis of Alas1 mRNA in the liver. (**B**) Western blotting analysis of Alas1 protein in the liver. Gapdh was used as a loading control. (**C**) Relative quantifications of Alas1 protein in the liver, based upon Western blotting. Data are expressed as mean ± SD (*n* = 4). The data in the control group were set as 1. **** *p* < 0.0001 vs. control.

The effects of PZA and INH on Alas1 expression were assessed at both the mRNA and protein levels. A qPCR analysis revealed no significant differences in Alas1 mRNA expression in either the PZA- or INH-treated groups compared to the controls (Figure 2A). In contrast, a Western blot analysis demonstrated a significant increase in Alas1 protein levels in the livers of the INH-treated mice, while the PZA treatment had no effect (Figure 2B,C). These findings indicate that INH, but not PZA, upregulates Alas1 post-transcriptionally, potentially disrupting the heme biosynthetic pathway.

### 3.3. Effects of PZA and INH on ALA Levels in the Liver and Urine

**Figure 3:**
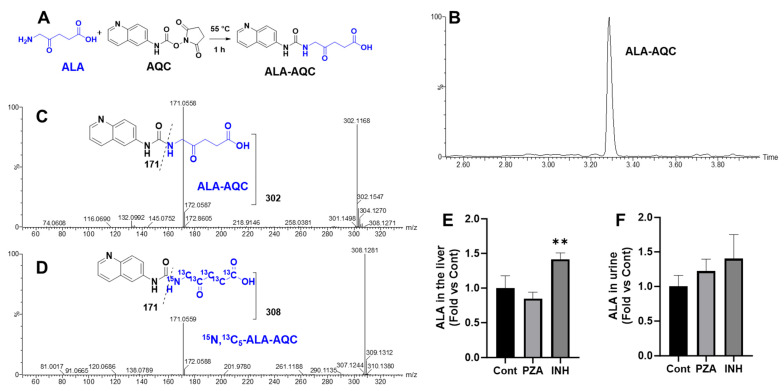
Identification and quantification of ALA in the liver and urine. (**A**) Trapping of ALA by AQC to form ALA-AQC. (**B**) UPLC spectrum of ALA-AQC. (**C**) MS/MS spectrum of ALA-AQC. (**D**) MS/MS spectrum of ^15^N, ^13^C_5_-ALA-AQC. (**E**) Quantification of ALA in the liver of mice treated with 2000 mg/L PZA or 400 mg/L INH in drinking water for 14 days. (**F**) Quantification of ALA in the urine of mice treated (po) with a single dose of 360 mg/kg PZA or 72 mg/kg INH. Data are expressed as mean ± SD (*n* = 4). The data in the control group were set as 1. ** *p* < 0.01 vs. control.

ALA, the product of ALAS1 activity [16], is a small molecule that cannot be directly detected by UPLC-QTOFMS. To enable detection, ALA was derivatized using 6-aminoquinolyl-N-hydroxysuccinimidyl carbamate (AQC) to form ALA-AQC (Figure 3A). The derivatized product displayed a retention time of 3.30 min (Figure 3B), and its identity was confirmed by MS/MS analysis, which revealed a characteristic fragment ion at *m*/*z* 171 (Figure 3C). The analytical method was further validated using a stable isotope-labeled internal standard, ^15^N*,*^13^C_5_-ALA (Figure 3D). Using this approach, ALA levels were quantified in the liver and urine samples from mice treated with PZA or INH. Compared to the controls, hepatic ALA levels were significantly elevated in the INH-treated group, while no significant change was observed in the PZA group (Figure 3E). Urinary ALA levels were also higher in the INH group, although the increase did not reach statistical significance (Figure 3F).

### 3.4. Effects of PZA and INH on Fech Expression in the Liver

**Figure 4:**
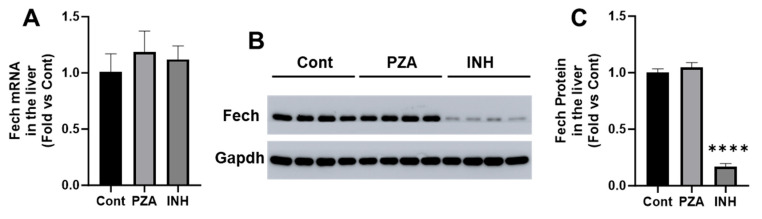
Effects of PZA and INH on Fech expression in the liver. WT mice were treated with PZA or INH for 14 days. (**A**) qPCR analysis of Fech mRNA in the liver. (**B**) Western blotting analysis of Fech protein in the liver. Gapdh was used as a loading control. (**C**) Relative quantifications of Fech protein in the liver, based upon Western blotting. Data are expressed as mean ± SD (*n* = 4). The data in the control group were set as 1. **** *p* < 0.0001 vs. control.

Next, we investigated the effects of PZA and INH on hepatic Fech expression. The qPCR analysis revealed no significant changes in the Fech mRNA levels in either the PZA- or INH-treated groups compared to the controls (Figure 4A). However, the Western blot analysis demonstrated a significant reduction in Fech protein levels in the livers of INH-treated mice, whereas PZA had no effect (Figure 4B,C). These findings suggest that INH downregulates Fech at the post-transcriptional level, potentially disrupting heme synthesis and leading to the accumulation of PPIX.

### 3.5. Effects of PZA and INH on PPIX Levels in the Liver and Feces

**Figure 5:**
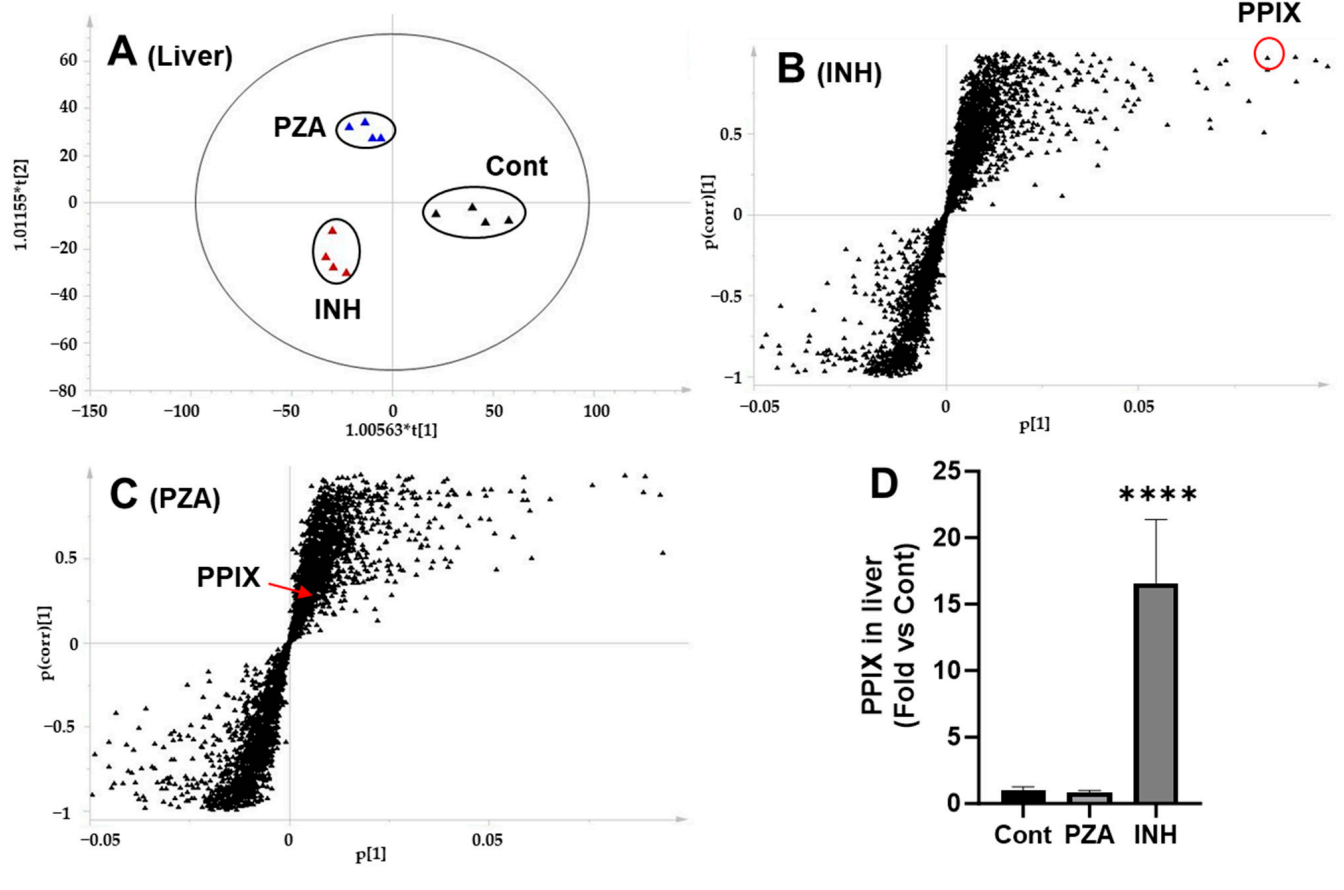
Identification and quantification of PPIX in the liver of mice treated with PZA or INH. (**A**) A score plot showing the separation of liver samples from the control, PZA, and INH groups. (**B**) A loading S-plot of the control and INH groups. PPIX was identified as one of the top-ranking ions in the liver of mice treated with INH. (**C**) A loading S-plot of the control and PZA groups showing PPIX as a low-ranking ion in the liver metabolome of mice treated with PZA. (**D**) Quantification of PPIX in the liver of mice treated with PZA or INH. Data are presented as mean ± SD (*n* = 4). The data in control group were set as 1. **** *p* < 0.0001 vs. control.

**Figure 6:**
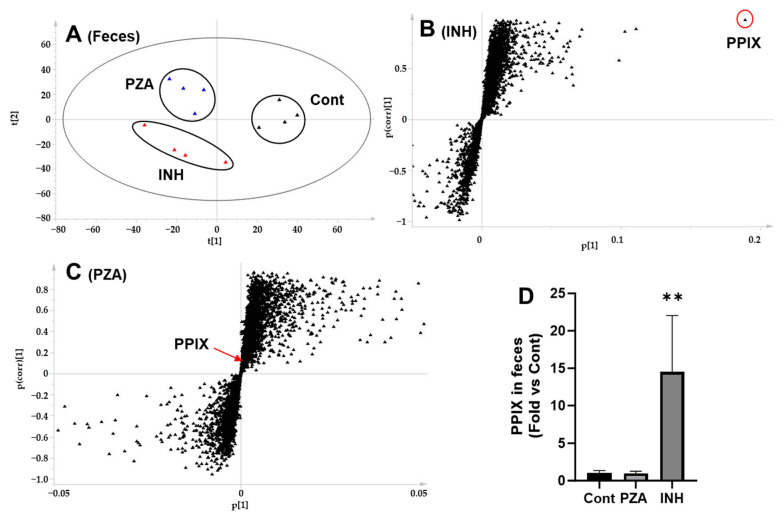
Identification and quantification of PPIX in the feces of mice treated with PZA or INH. (**A**) A score plot showing the separation of fecal samples from the control, PZA, and INH groups. (**B**) A loading S-plot of the control and INH groups. PPIX was identified as the number 1 top-ranking ion in the feces of mice treated with INH. (**C**) A loading S-plot of the control and PZA groups showing PPIX as a low-ranking ion in the fecal metabolome of mice treated with PZA. (**D**) Quantification of PPIX in the feces of mice treated with PZA or INH. Data are presented as mean ± SD (*n* = 4). The data in the control group were set as 1. ** *p* < 0.01 vs. control.

PPIX is the substrate of FECH [17]. To assess the impact of PZA and INH on Fech function, we measured the PPIX levels in the mouse liver and fecal samples. A PCA analysis of the liver metabolomic profiles revealed clear separation among the control, PZA-, and INH-treated groups (Figure 5A). Furthermore, S-plots generated from the OPLS-DA analysis identified the ions responsible for the group separation (Figure 5B,C). PPIX was prominently featured in the upper right quadrant of the S-plot for the INH group, indicating its elevated abundance (Figure 5B), whereas no such enrichment was observed in the PZA group. Consistent with these analyses, direct quantification confirmed a significant increase in the hepatic PPIX levels following INH treatment, while PZA had no effect (Figure 5D). Similar trends were observed in the fecal samples as PPIX levels were significantly elevated in the INH group compared to that in the controls, whereas the PZA group exhibited no significant difference (Figure 6).

### 3.6. Effects of PZA and INH on Liver Function

Neither PZA nor INH treatment resulted in significant changes in serum ALT, AST, or ALP levels compared to the control group (Supplementary Figure S2A–C). In addition, the histological analysis revealed no observable liver damage in either the PZA- or INH-treated groups (Supplementary Figure S2D–F). These findings suggest that INH alone does not induce significant liver injury in mice, despite its ability to cause PPIX accumulation in the liver.

## 4. Discussion

**Figure 7:**
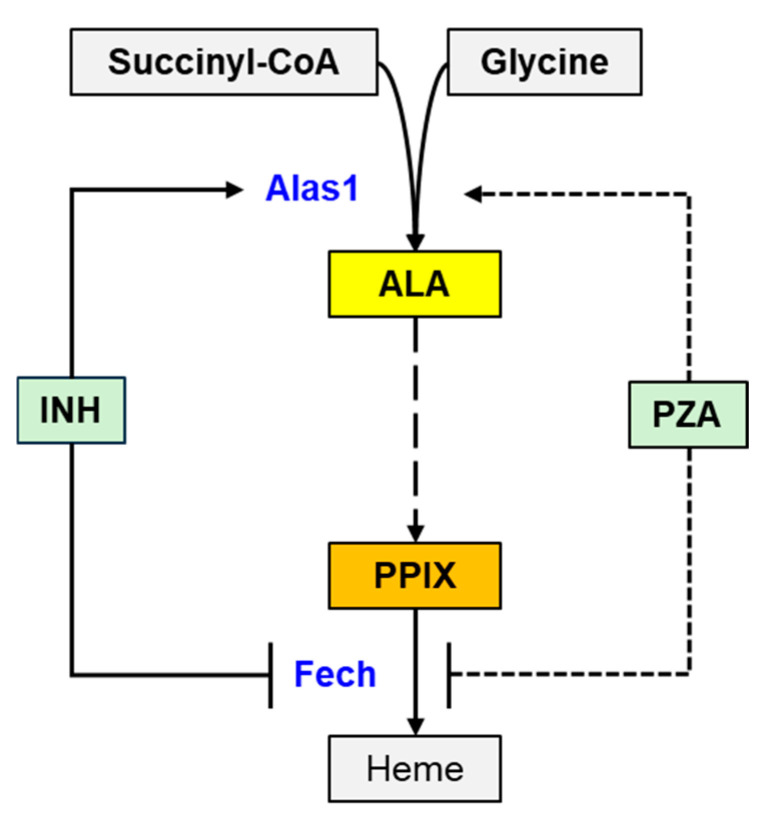
A schematic illustrating the effects of INH and PZA on the expression and function of Alas1 and Fech in mouse liver. INH upregulates Alas1, leading to the increased production of ALA. Additionally, INH downregulates Fech, resulting in the accumulation of PPIX. In contrast, PZA does not affect the expression or function of either Alas1 or Fech in mouse liver.

In this study, we performed a systematic comparison of the effects of INH and PZA, two first-line anti-TB drugs, on the heme biosynthetic pathway in mouse liver. Our investigation was motivated by the fact that both drugs are currently contraindicated in porphyria [9,10,11,12,13], a group of disorders arising from defects in heme biosynthesis. Despite these clinical cautions, the mechanisms underlying these contraindications, particularly for PZA, have remained insufficiently understood. The current work demonstrated that INH, but not PZA, significantly perturbs heme biosynthesis by altering the expression and activity of key enzymes, Alas1 and Fech, leading to the accumulation of toxic intermediates including ALA and PPIX (Figure 7). These findings provide a mechanistic basis for the contraindication of INH in porphyria and suggest that the risk associated with PZA may have been previously overestimated, warranting further investigation.

The heme biosynthetic pathway is tightly regulated, particularly at two key enzymatic steps: the initial condensation of glycine and succinyl-CoA by ALAS1, and the final insertion of ferrous iron into PPIX by FECH [15,26]. Disruptions at either point can lead to the accumulation of pathway intermediates, triggering porphyric attacks [15,27]. Our data showed that INH treatment in mice significantly increases Alas1 protein levels in the liver without affecting mRNA expression, suggesting that INH may enhance Alas1 protein stability or interfere with its degradation pathways, leading to elevated enzyme levels and increased ALA synthesis. Consistently, we observed elevated ALA concentrations in the liver of mice treated with INH.

Simultaneously, INH reduced the hepatic Fech protein levels in mouse liver, again without affecting mRNA expression. This suggests that INH might either promote Fech degradation or inhibit its translation, though the precise mechanism remains to be elucidated. The INH-mediated downregulation of Fech impairs the conversion of PPIX to heme, resulting in PPIX accumulation in mouse liver and feces. These data are consistent with previous reports linking INH to porphyria exacerbation and provide a more detailed mechanistic framework for this interaction [18,19]. Together, the upregulation of Alas1 and downregulation of Fech by INH synergistically shift the metabolic balance toward the accumulation of porphyrin intermediates. This dual-hit effect significantly elevates the risk for porphyria in susceptible individuals and strongly supports the classification of INH as a high-risk drug for porphyria exacerbation.

Our work demonstrates that the INH-mediated regulation of ALAS1 and FECH proteins does not occur at the transcriptional level. CYP2E1, a cytochrome P450 enzyme primarily expressed in the liver, plays a crucial role in the metabolism of various compounds [28]. A similar regulatory pattern has been observed for CYP2E1, where INH-induced increases in CYP2E1 protein levels were not accompanied by corresponding changes in mRNA expression [29]. Mechanistically, studies have shown that CYP2E1 expression in the liver is regulated at the post-transcriptional or post-translational levels, either through mRNA stabilization or by protection against rapid protein degradation [30,31]. Further research is warranted to elucidate the mechanisms by which INH regulates ALAS1 and FECH protein levels.

In contrast to INH, PZA had no significant effect on Alas1 or Fech expression at either the mRNA or protein level. Consistently, no changes were observed in the hepatic or excretory levels of ALA or PPIX in mice treated with PZA. These findings indicate that PZA does not disrupt the heme biosynthetic pathway. Our results differ from earlier studies from the 1960s and 1970s, which reported that PZA may induce Alas activity [20,21]. However, those studies were limited to in vitro experiments or in vivo models involving a single dose of PZA [20,21]. Additionally, they relied on less sensitive analytical techniques and did not directly assess the mRNA expression, protein levels, or enzymatic activity of Alas1. In contrast, our study employed a subchronic treatment model and utilized quantitative methods, including qPCR, Western blotting, and UPLC-QTOFMS, to comprehensively evaluate the effects of PZA. Our data suggest that the current classification of PZA as contraindicated in porphyria may warrant re-evaluation. In addition, species-specific differences in response to PZA in the heme biosynthetic pathway should also be taken into account.

The clinical implications of our findings are significant. TB remains a major global health challenge, and treatment typically involves a combination of first-line drugs, including INH and PZA [32]. However, the current contraindications for these drugs severely limit treatment options for TB patients with porphyria. Our results suggest that INH warrants cautious use in patients with porphyria because it may cause the accumulation of heme intermediates by regulating Alas1 and Fech. In contrast, despite historical concerns, PZA does not appear to disrupt the heme biosynthetic pathway in mice and may therefore be considered a safe therapeutic option for patients with porphyria. These findings are particularly relevant for clinicians treating TB in porphyria patients and for regulatory bodies responsible for drug safety.

In summary, this study elucidates the distinct effects of INH and PZA on the heme biosynthetic pathway in the mouse liver. INH induces ALAS1 protein expression while suppressing FECH, leading to the accumulation of ALA and PPIX and thereby increasing the risk of porphyria. In contrast, PZA does not significantly alter the expression or activity of ALAS1 or FECH and does not result in the buildup of porphyrin precursors. These findings provide novel insights into the biochemical mechanisms underlying porphyria risk during anti-tuberculosis therapy and support a re-evaluation of the current contraindication of PZA in patients with porphyria. We also recommend that future studies investigate the molecular mechanisms by which INH regulates ALAS and FECH, as well as the long-term effects of PZA, INH, and their metabolites on the heme biosynthetic pathway.

## Data Availability

The data are contained within this article.

## Supplementary Materials

The following supporting information can be downloaded at: https://www.mdpi.com/article/10.3390/metabo15060355/s1. Supplementary Figure S1. Detection of PA in the serum of mice treated with PZA. Supplementary Figure S2. Evaluation of liver injury in mice treated with PZA or INH.

## Abbreviations

The following abbreviations are used in this manuscript:

ALA	Aminolevulinic acid
ALAS	Aminolevulinic acid synthase
ALT	Alanine transaminase
ALP	Alkaline phosphatase
AQC	6-Aminoquinolyl-N-hydroxysuccinimidyl carbamate
AST	Aspartate transaminase
FECH	Ferrochelatase
GADPH	Glyceraldehyde 3-phosphate dehydrogenase
INH	Isoniazid
^15^N,^13^C_5_-ALA	^15^N,^13^C_5_-Aminolevulinic acid
OPLS-DA	Orthogonal partial least-squares discriminant analysis
PA	Pyrazinoic acid
PCA	Principal component analysis
PPIX	Protoporphyrin IX
PZA	Pyrazinamide
QTOFMS	Quadrupole time of flight mass spectrometry
UPLC	Ultra-performance liquid chromatography
TB	Tuberculosis
