# Supplementary material for: Comparison of Pyrazinamide with Isoniazid for Their Effects on the Heme Biosynthetic Pathway in Mouse Liver
Source: Metabolites. 2025 May 28;15(6):355. doi: 10.3390/metabo15060355 (PMC12195290; doi:10.3390/metabo15060355)
Supplement: Supplementary file 1 [file metabolites-15-00355-s001.zip › metabolites-3645643-supplementary.pdf]

*SUPPLEMENTARY MATERIALS*

# Comparison of Pyrazinamide with Isoniazid for Their Effects on the Heme Biosynthetic Pathway in Mouse Liver

**Fu-Ying Qin, Ruizhi Gu, Jiaojiao Zhang, Jaden Leigh Weiss, Jie Lu, Qing Ma, and Xiaochao Ma**

**Supplementary Figure S1. Detection of PA in the serum of mice treated with PZA.**

**Supplementary Figure S2. Evaluation of liver injury in mice treated with PZA or INH.**

## Supplementary Figure S1

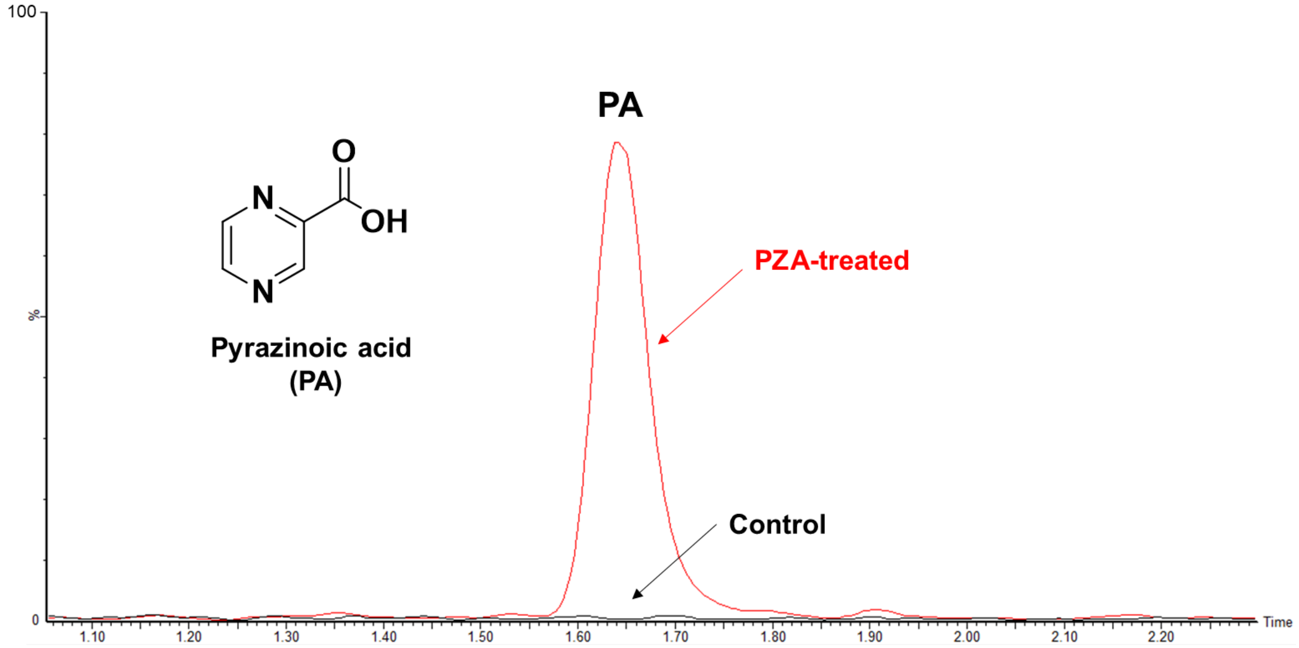


**Supplementary Figure S1. Detection of PA in the serum of mice treated with PZA.** Wild-type mice were treated with PZA for 14 days. PA was detected by UPLC-QTOFMS operated in negative mode.

**Supplementary Figure S2**

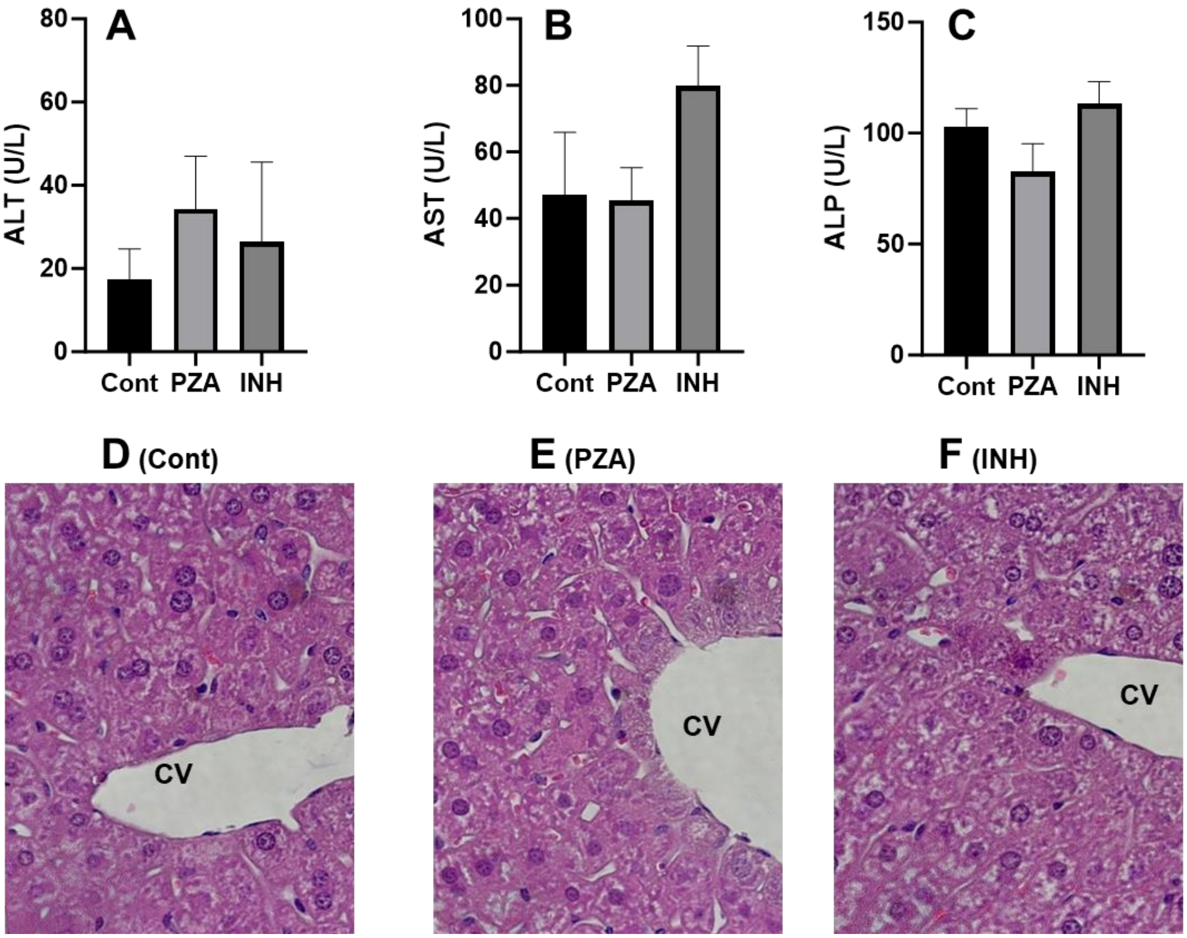


**Supplementary Figure S2. Evaluation of liver injury in mice treated with PZA or INH.** WT mice were treated with PZA or INH 14 days. (**A-C**) Serum activities of ALT, AST, and ALP. Data are expressed as mean ± SD (n = 4). (**D-F**) Histological analysis of liver sections. Liver sections were stained with hematoxylin and eosin. CV, central vein.
